# Association Between Systemic Immune-Inflammatory Index (SII) and Cancer Grading and Staging: Evidence from the Current Scientific Literature

**DOI:** 10.3390/biology15030253

**Published:** 2026-01-30

**Authors:** Alessandro Rizzo, Elsa Vitale, Lorenza Maistrello, Kazuki Santa, Matteo Santoni

**Affiliations:** 1 S.S.D. C.O.r.O. Bed Management Presa in Carico, TDM, IRCCS Istituto Tumori “Giovanni Paolo II”, Viale Orazio Flacco 65, 70124 Bari, Italy; rizzo.alessandro179@gmail.com; 2 Directorate of Health Professions and Nursing, ASL Bari, 70123 Bari, Italy; 3 IRCCS San Camillo Hospital, 30126 Venice, Italy; lorenza.maistrello@hsancamillo.it; 4 Faculty of Medical Science, Juntendo University, Urayasu, Chiba 279-0013, Japan; k-santa@juntendo.ac.jp; 5 Department of Biotechnology, Tokyo College of Biotechnology, Ota-ku, Tokyo 144-0032, Japan; 6 Oncology Unit, Macerata Hospital, 62100 Macerata, Italy; matteo.santoni82@gmail.com

**Keywords:** grading, neoplasms, staging, systemic inflammation index

## Abstract

Background: Accumulating evidence demonstrates that inflammatory responses play a critical role in tumor progression, invasion, and metastasis. In recent years, systemic immune-inflammatory (SII) markers have emerged as valuable tools for evaluating tumor grade and prognosis in solid tumors. To assess SII scores associated with neoplasm-related grading and staging in order to investigate the incidence rate in SII levels and cancer-related aggressiveness. Methods: The present review was recorded in Figshare system with id no. 10.6084/m9.figshare.31029121. Observational studies recording grading and staging differences (advanced III–IV vs. early 0–II) in SII values among cancer patients were included in the present review. Frequencies were collected among cancer patients, along with low and high SII levels and related grading and staging scores. Results: A total of ten studies were included in the present review. Considering cancer grading classification and SII levels, our data showed significant associations not recorded in all the selected studies. Additionally, high SII values appeared to be significantly associated with advanced-stage cancer (stages III–IV), while lower SII values were associated with an increased probability of early-stage tumors (stages 0–II), (*p* < 0.05). Conclusions: The SII marker has shown potential predictive value in cancer. However, the SII is a relatively novel index in which a direct causal effect on tumor initiation or subsequent disease progression remains to be comprehensively investigated.

## Simple Summary

*Staging* indicates the size of the primary tumor, whether it has involved nearby lymph nodes, and whether it has spread (metastasized) to other parts of the body, often using categories ranging from stage I (localized disease) to stage IV (metastatic disease), whereas *grading* assesses the aggressiveness of tumor cells based on their microscopic appearance; together, these parameters help guide treatment planning. Accumulating evidence demonstrates that inflammatory responses play a critical role in tumor progression, invasion, and metastasis. In recent years, systemic immune-inflammatory (SII) markers have emerged as valuable tools for evaluating tumor grade and prognosis in solid tumors. To assess SII scores associated with neoplasm-related grading in order to investigate the incidence rate in SII levels and cancer-related aggressiveness. The SII marker has showed potential predictive value in cancer. However, the SII is a relatively novel index in which a direct causal effect on tumor initiation or subsequent disease progression remains to be comprehensively investigated.

## 1. Introduction

In 1979, the World Health Organization (WHO) started to promote the tumor grading system, successively updated in 1993, 2000, 2007, and 2016 [1,2]. The WHO classification and grading of cancers are initially assessed on histopathological features improved by immunohistochemical (IHC) examination of tumor signals and expansion indices [3]. Cancers are associated grades from WHO grade I to IV according to the extension of anaplastic peculiarities. WHO grade I suggests indolent tumors that are often associated with encouraging clinical prognoses, whereas WHO grade IV indicates highly aggressive malignancies with unfavorable prognoses [4]. In particular, grade I tumors are slow-development, nonmalignant, and linked to long-term survival; grade II tumors display cytological atypia and slow improvement, being either benign or malignant in nature; grade III tumors show anaplasia and increased mitotic activity, indicating malignancy and typically higher-grade features; and grade IV tumors suggest three or four malignancy criteria, like anaplasia, high mitotic activity, microvascular growth, and/or necrosis with high growth and excessive aggressiveness as well [5,6].

This grading system helps oncologists in assessing tumor recurrence risk and prognosis. Assessing the aggressiveness of neoplasms is essential for directing treatment approaches, as the recognition of cost-effective and predictable prognostic biomarkers can give information on therapeutic decision-making and improvements in clinical outcomes.

Commonly used prognostic factors include AJCC stage, pathological grade, and histological classification. However, these parameters have notable limitations, including limited predictive accuracy, high costs, challenges in detection, and delays in obtaining results [7].

The Eighth Edition of the AJCC Cancer Staging Manual, published in October 2016, embraces all currently available information on the staging of adult cancers for all clinically important anatomic sites, which also includes the anatomic extent of disease tumor, lymph node, and metastasis (TNM) principles that were first developed by Pierre Denoix in the 1940s and 1950s [8].

The linkage between tumors and inflammation has been studied since the 19th century, when Rudolf Virchow explained that cancer can develop from sites of chronic inflammation [9,10,11]. Nowadays, evidence try to explain the complex association between inflammation and cancer [12,13,14], suggesting two main pathways involved in this association as the molecular and cellular processes. The intrinsic mechanism is guided by genetic dysfunctions that induce tumorigenesis followed by inflammation-related processes creating the tumor inflammatory microenvironment. On the other hand, the extrinsic mechanism includes inflammatory features that improve carcinogenesis and progression [15].

Additional evidence shows that inflammatory responses have an essential role in tumor growth, development, and metastasis [16,17,18]. Thus, considering inflammation-related indices into cancer evaluation may ameliorate the accurate prediction of tumor biology and patient prognosis. In recent years, systemic immune-inflammatory (SII) markers have been recognized as useful instruments for assessing tumor grade and prognosis in solid tumors [19]. In particular, the SII is an innovative biomarker that has been recognized as an independent predictor of poor prognosis in numerous cancer types [20,21].

The SII was firstly assessed by Hu et al. in 2014 [22], who performed the formula: SII = (platelet (P) × blood neutrophil (N))/lymphocyte (L). The SII has been showed to be a powerful prognostic index of poor clinical outcomes [23]. The idea that higher SII values may predict tumor grade and biological aggressiveness provides its potential usage in clinical oncology settings [24,25,26].

In light of what was explained above, the present review aimed to assess SII scores associated with neoplasms-related grading and staging in order to investigate the incidence rate in SII levels and cancer-related aggressiveness.

## 2. Materials and Methods

### 2.1. Searching Approach

The present literature review was recorded in Figshare system (https://info.figshare.com/) with id no. 10.6084/m9.figshare.31029121. All observational studies recording grading and staging differences (advanced III–IV vs. early 0–II) in SII levels among cancer patients were included in the present review. Frequencies were collected among cancer patients, and low and high SII levels, as well related grading and staging scores, were classified as “advanced” III–IV and “early” 0–II.

**Figure 1:**
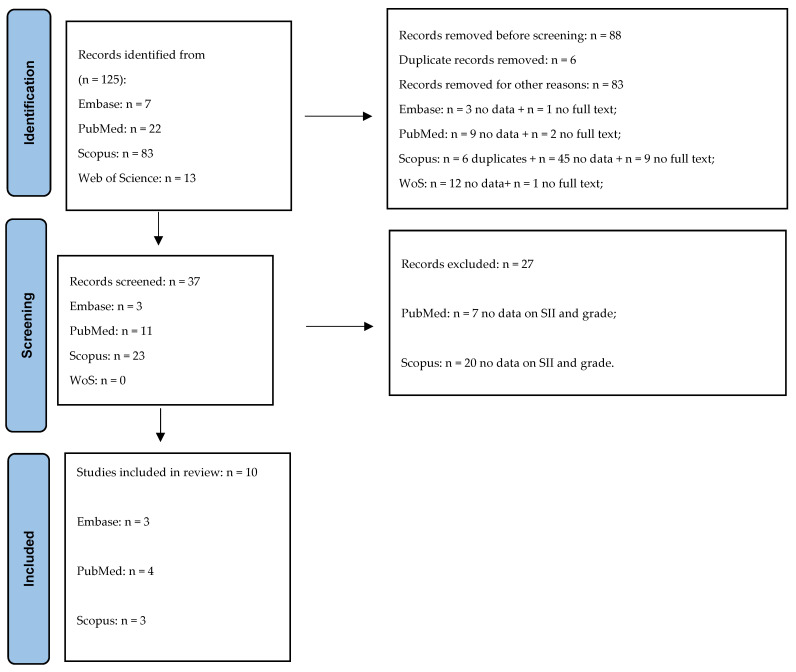
The PRISMA flow chart for the study selection process.

Keywords used to perform the present literature review were as follows: “neoplasm grading” and “systemic inflammation index” (Supplementary File S1), and the literature research was performed in Embase, PubMed, Scopus, and Web of Science databases. Literature screening was performed according to the PRISMA flow chart [27] (Figure 1). Initially, we found a total of 125 records. Of these, 89 records were excluded and then, from the remained 36 records, other 27 were further excluded since they did not contain SII-related data associated with neoplasm grading. Finally, a total of 9 records were identified belonging to Embase (*n* = 2), PubMed (*n* = 4), and Scopus (*n* = 3).

### 2.2. Main Outcome(s)

Association between high vs. low SII and both tumor grade and stage (advanced III–IV vs. early 0–II) was found.

### 2.3. Data Extraction

Initially, records were identified through a systematic database search and uploaded to a reference management software where duplicate studies were removed. Then, two independent reviewers (E.V. & L.M.) assessed the title and abstract of the identified studies for inclusion, and unsuitable reports were removed. After that, articles were uploaded, and the full text was assessed more closely for eligibility. Disagreements about whether a study should be included or not were resolved by discussion and consensus. If the disagreement remained, arbitration from another reviewer was provided (A.R.). Data collection was extracted by considering study characteristics (author, year of publication, aim, design, sample size, setting), participants (age, cancer grade and stage), and proportion of cases of stage- and grade-related cancers were collected according to the assessed SII levels.

### 2.4. Quality Assessment and Risk of Bias

The studies were assessed for quality as per protocol recommendations. The information collected from the final screened studies were explained adopting a narrative approach.

The quality assessment of all the included studies was performed by considering their study designs and related levels of evidence according to the evidence-based nursing (EBN) approach [28]. The EBN strategy embraced a total of seven levels of evidence, ranging from I to VII, suggesting the weakest quality of study design, specifically the following:

1. Level I: Evidence from reviews or meta-analysis of randomized control trials;
2. Level II: Evidence from well-designed randomized control trials;
3. Level III: Evidence from well-designed control trials that are not randomized;
4. Level IV: Evidence from case–control or cohort studies;
5. Level V: Evidence from reviews of descriptive or qualitative studies;
6. Level VI: Evidence from a single descriptive or qualitative study;
7. Level VII: Evidence from expert opinions.

In the present literature review, we included all studies belonging from I to VI levels of evidence.

### 2.5. Strategy for Data Synthesis

The primary objective of this literature review was to investigate whether there was an association between the systemic immune-inflammation index (SII), categorized as high or low according to study-specific cut-off values, and both tumor grade and stage, dichotomized as early stage (0–II) versus advanced stage (III–IV).

## 3. Results

### Selected Studies

At the beginning of our literature review we collected a total of 125 records searching in Embase (*n* = 7), PubMed (*n* = 22), Scopus (*n* = 83), and Web of Science (*n* = 13) databases (Figure 1). Before screening, we excluded 89 records, since most of them did not present data according to grade, stage, and SII levels, or they did not contain the full text of the manuscript. Then, an additional 27 studies were excluded, since data did not explain our literature review aim. Then, a total of 10 studies that highlighted grading or staging assessment according to SII levels were included as high or low [22,29,30,31,32,33,34,35,36,37]. Among the selected studies, three studies [22,29,33] explained both assessments in cancer-related grading and staging according to SII levels. Table 1 showed the main features in the screened studies regarding grading and SII assessments.

Table 2 showed the main features in the screened studies regarding staging and SII assessment.

## 4. Discussion

Considering cancer grading classification and SII levels, our data showed significant associations recorded not in all the selected studies. In fact, in vulvar cancer [29], in hepatocellular carcinoma [22], in epithelial ovarian cancer [33], and in endometrial cancer [34], we assessed insignificant associations between cancer-related grading and SII levels. In hepatocellular carcinoma [22], we recorded a significant association in the validation cohort group, who underwent resection and were then prospectively recruited (*p* = 0.021). On the other hand, in the cohort group, the association between grading score and SII levels was not statistically significant (*p* = 0.198).

Conversely, our data collected showed that elevated SII values appeared to be significantly associated with advanced-stage cancer (stages III–IV), while lower SII values were associated with an increased probability of early-stage tumors (stages 0–II). In fact, in all the included studies assessing cancer-related stages, significant associations were recorded between cancer stages and SII levels (*p* < 0.05). This trend was not explained only in Farolfi et al.’s study [35], in which associations in SII levels and cancer stages were only investigated between high staging levels, like II and IV, and not between low and high levels. On the other hand, in all the other selected studies, associations between low and high stages and SII levels were all statistically significant for all the cancer typology analyzed. These significant associations underlined the complex interplay between the SII and tumor development. Higher SII levels could improve immune evasion, hinder apoptotic mechanisms, and accelerate genomic vulnerability, angiogenesis, spread, and metastatic malignancies [38,39,40]. Neutrophils, lymphocytes, and monocytes were involved in carcinogenesis and growth through their functions in the systemic inflammatory response [41].

The prognostic value of SII has also been investigated in malignancies with peritoneal spread. Yan et al. [41] reported that higher preoperative neutrophil-to-lymphocyte (N–L), platelet-to-lymphocyte (P–L), and SII scores were associated with more unfavorable overall survival. Additionally, low SII seemed to be the only helpful prognostic index [42]. However, the SII could reflect the balance between host inflammatory condition and immune response in cancer patients with less confounding factors [35], since cancer-related systemic inflammation often anteceded both tumor proliferation and inflammatory biomarkers as well [43,44]. For example, thrombocytosis, often persisting in patients suffering from epithelial ovarian cancer (EOC), has been connected with unfavorable prognosis contributing to epithelial–mesenchymal shift in circulating tumor cells, improving metastatic spread [45,46]. At the same time, lymphocytes appeared to have an essential role in antitumor immunity by enhancing cytotoxic tumor cell death and preventing tumor cell proliferation and migration [47,48].

Higher SII values have been associated with unfavorable clinical outcomes in several malignancies, like cervical, gastrointestinal, pancreatic, and bladder malignancies [49]. In this regard, a meta-analysis displayed a significant association between higher SII levels and poor prognosis in glioma, accompanied by decreased overall survival (OS) and progression-free survival (PFS) values [50]. On the other hand, additional evidence [51] did not recognize a significant association between SII and cancer outcomes. Geraghty et al. [50] described SII as an innovative and readily available biomarker that seemed to be independently correlated with mitotic activity, serving as a predictor of tumor grade [52,53].

The prognostic role played by SII could be explained by the important role of inflammation and immune regulation in creating tumor biological patterns [54,55]. This relationship has been suggested as an indication in cancer [56]. Moreover, platelets have been recognized as protective factors in tumor cells from immune-mediated cytolysis, which also improve tumor progression through platelet-derived intermediaries and inflammatory cytokines [57,58]. Neutrophils applied tumor-enhancing effects by stimulating an immunosuppressive microenvironment, whereas lymphocytes support T-cell-mediated antitumor immunity [59]. All these functions could address the profound relationships between high SII values and high-grade malignancies [43,60].

Thus, current scientific evidence suggested the idea that systemic inflammation, quantified thanks to the SII, could reliably serve as an indicator of tumor aggressiveness and unfavorable prognosis [61].

### Strengths and Limitations

The present literature review addressed the association between SII and tumor grading and staging across multiple cancer types. Surely, the topic could represent a clinically relevant one and be of broad interest to oncology and cancer biology researchers. However, differences in SII cut-off values among all the included studies, cancer-specific grading systems, and treatment-related confounders should be considered. Thus, the present literature review could be a pilot study that aimed to summarize and address the main findings in SII levels and grading and staging cancers. However, a more cautious interpretation of the pooled effect size would be advisable given the moderate-to-high heterogeneity observed.

## 5. Conclusions

The association between inflammation and cancer appeared to be exhaustively displayed, although it remained partially investigated due to its elaborate and multifactorial essence [62,63]. Inflammation has been thought to be actively involved in carcinogenesis or, conversely, it could represent a systemic response to an undetected tumor or to DNA damage, suggesting potential prognostic biomarkers [64,65].

However, the literature has just suggested that the SII could represent a real, non-invasive, and easily predictive biomarker indicating aggression level in cancer patients.

However, the literature suggested that a single marker could not exhaustively predict prognostic power while also classifying patients according to their risk and personalized treatment approaches. However, the SII could be introduced in a monitoring protocol to better understand individual patient risk. Adopting the SII in clinical practice could be helpful in identifying high-risk patients early and adopt targeted interventions and preventive approaches [66].

## Figures and Tables

**Table 1:** Features of the included studies on grading and SII assessment (*n* = 7).

Author(s) Publication Year	Study Design	Cancer Typology	Grading Assessment	Patients No.	SII Ref. Values	SII_Low	SII_High	Cr_Grade 0–II_ SII_Low	Cr_Grade III–IV_ SII_Low	Cr_Grade 0–II_ SII_High	Cr_Grade III–IV_ SII_High	*p*-Value
Bartl et al., 2021 [29]	Retrospective study	Invasive vulvar cancer	Histologic grading	130	866.4 < SII ≥ 866.4	65	65	55	10	53	12	0.230
Hu et al., 2014 [22]	Retrospective study	Hepatocellular Carcinoma	Edmondson grading system	133	330 < SII ≥ 330	80	53	61	35	19	18	0.198
Hu et al., 2014 [22]	Retrospective study	Hepatocellular Carcinoma	Edmondson grading system	123	330 < SII ≥ 330	82	41	61	22	21	19	0.021 *
Jiang et al., 2025 [30]	Retrospective study	Renal cell carcinoma	Fuhrman grade	240	849.48 < SII ≥ 849.48	170	70	150	20	53	17	0.025 *
Liang et al., 2018 [31]	Retrospective study	Gliomas	WHO grade	153	392.48 < SII ≥ 392.48	60	93	35	25	18	75	<0.001 *
Liang et al., 2019 [32]	Retrospective study	Gliomas	WHO grade	169	324.38 < SII ≥ 324.38	53	116	0	53	0	116	0.009 *
Nie et al., 2019 [33]	Retrospective study	Epithelial ovarian cancer	Tumor grade	250	612 < SII ≥ 612	129	121	35	94	31	90	0.886
Nie et al., 2019 [33]	Retrospective study	Epithelial ovarian cancer	Tumor grade	283	612 < SII ≥ 612	135	148	38	97	41	107	0.934
Njoku et al., 2022 [34]	Prospective study	Endometrial cancer	Tumor grade and histology	367	910 < SII ≥ 910	299	168	221	107	78	61	0.065

*Abbreviations*: SII, Systemic Immune-Inflammation Index; WHO, World Health Organization. * *p* < 0.05 is statistical significant.

**Table 2:** Features of the included studies in staging and SII assessment (*n* = 7).

Author(s) Publication Year	Study Design	Cancer Typology	Staging Assessment	Patients No.	SII Ref. Values	SII_Low	SII_High	Cr_Stage 0–II_ SII_Low	Cr_Stage III–IV_ SII_Low	Cr_Stage 0–II_ SII_High	Cr_Stage III–IV_ SII_High	*p*-Value
Bartl et al., 2021 [29]	Retrospective study	Invasive vulvar cancer	FIGO stage	130	866.4 < SII ≥ 866.4	65	65	48	17	38	27	0.029 *
Farolfi et al., 2020 [35]	Observational, Multicenter study	Recurrent epithelial ovarian cancer patients	FIGO stage	375	730 < SII ≥ 730	156	219	156	---	---	219	0.074
Jiang et al., 2025 [30]	Retrospective study	Renal cell carcinoma	AJCC stage	240	849.48 < SII ≥ 849.48	170	70	129	41	35	35	<0.001 *
Nie et al., 2019 [33]	Retrospective study	Epithelial ovarian cancer	FIGO stage	250	612 < SII ≥ 612	129	121	46	83	24	97	0.007 *
Nie et al., 2019 [33]	Retrospective study	Epithelial ovarian cancer	FIGO stage	283	612 < SII ≥ 612	135	148	37	98	21	127	0.006 *
Njoku et al., 2022 [34]	Prospective study	Endometrial cancer	FIGO-stage	367	910 < SII ≥ 910	327	139	221	107	78	61	0.024 *
Shi et al., 2018 [36]	Retrospective study	Gastric cancer	TNM stage (AJCC, 8th)	688	320 < SII ≥ 320	356	332	286	41	107	32	<0.001 *
Shi et al., 2018 [36]	Retrospective study	Gastric cancer	TNM stage (AJCC, 8th)	174	320 < SII ≥ 320	90	84	61	9	70	34	<0.001 *
Zhang et al., 2020 [37]	Retrospective study	Locally advanced rectal cancer	TNM stage	472	230 < SII ≥ 230	309	163	227	82	111	57	<0.001 *

*Abbreviations*: FIGO, International Federation of Gynecology and Obstetrics; SII, Systemic Immune-Inflammation Index; TNM, Tumor, Node, Metastasis stage. * *p* < 0.05 is statistical significant.

## Data Availability

Data are available upon reasonable request from the corresponding author.

## Supplementary Materials

The following supporting information can be downloaded at https://www.mdpi.com/article/10.3390/biology15030253/s1; Supplementary File S1: Search strings carried out to perform this literature review. Supplementary File S2: Preferred Reporting Items for Systematic reviews and Meta-Analyses extension for Scoping Reviews (PRISMA-ScR) Checklist. Reference [67] is cited in the Supplementary Materials.
